# Application of a new composite genetic marker semen-specific methylation-microhaplotype in the analysis of semen–vaginal fluid mixtures

**DOI:** 10.1098/rsos.241565

**Published:** 2025-01-15

**Authors:** Dan Wen, Hao Xing, Xuan Tang, Yue Wang, Bowei Jiang, Jienan Li, Ying Liu, Lagabaiyila Zha

**Affiliations:** ^1^ Department of Forensic Medicine, School of Basic Medical Sciences, Central South University, No172. Tongzipo Road, Changsha, Hunan 410013, People’s Republic of China; ^2^ The First Research Institute of the Ministry of Public Security of P.R.C, No.1. Shouti South Road, Haidian District, Beijing 100044, People’s Republic of China; ^3^ Department of Oral Implantology, Xiangya Hospital of Stomatology, Central South University, No72. Xiangya Road, Changsha, Hunan 410028, People’s Republic of China

**Keywords:** forensic genetics, DNA methylation, microhaplotype, semen–vaginal fluid mixtures, K-nearest neighbour algorithm, massively parallel sequencing

## Abstract

DNA mixtures containing semen and vaginal fluid are common biological samples in forensic analysis. However, the analysis of semen–vaginal fluid mixtures remains challenging. In this study, to solve these problems, it is proposed to combine semen-specific CpG sites and closely related microhaplotype sites to form a new composite genetic marker (semen-specific methylation-microhaplotype). Six methylation-microhaplotype loci were selected. To further improve discrimination power, five methylation-SNP loci were also included. The methylation levels and genotypes of these selected loci were obtained using massively parallel sequencing technology. Except for loci MMH04ZHA019 and MMH17ZHA059, the remaining nine loci were successfully sequenced. For the successfully sequenced loci, they performed well in identifying individuals and body fluids. An allele categorization model was developed using K-nearest neighbour algorithm, which was then used to predict allele types in semen–vaginal fluid mixtures. These loci were able to confirm the presence of semen and link semen to a true donor in semen–vaginal fluid mixtures with mixing ratios of 10:1, 9:1, 5:1, 4:1, 1:1, 1:3, 1:4, 1:8 and 1:9 (semen:vaginal fluid). This preliminary study suggests that this new composite genetic marker has great potential as a supplementary tool to commonly used genetic markers (STR, etc.) for analysing semen–vaginal fluid mixtures.

## 1. Introduction

DNA mixtures are common biological samples in forensic analysis, consisting of DNA patterns from two or more contributors. A mixture (semen–vaginal fluid mixture) consisting of semen and vaginal fluid is usually obtained from sexual assault cases [1]. For the investigation of semen–vaginal fluid mixtures, it is important to link semen to a true donor. However, when body fluid identification is omitted in mixture analysis, false inferences may occur in forensic investigations. As proposed by Gill [2], these phenomena are known as ‘association fallacy’, which may seriously interfere with forensic investigations [2]. Therefore, it is necessary to simultaneously confirm the presence of semen and link semen to a true donor in semen–vaginal fluid mixtures.

Common body fluid identification methods include enzymatic, immunologic, mRNA/miRNA, bacteria and DNA methylation tests [3–7]. DNA methylation has attracted more and more attention due to its stability, body fluid specificity and compatibility with traditional DNA genotyping. However, in body fluid mixtures, it becomes difficult to confirm the presence of target body fluid using DNA methylation tests, and it is not possible to use DNA methylation tests to obtain the genotypes of target body fluid-specific DNA. To genotype the semen-specific DNA, differential extraction strategy and laser capture microdissection technology are commonly used to separate semen components [8,9]. But these two methods are not suitable for detection of mixtures containing small amounts of semen or no intact cells. Detection of genetic markers within the Y chromosome is another strategy for obtaining semen-specific information, but it is not possible to distinguish between individuals from the same paternal lineage [10].

Recently, a series of novel genetic markers have been proposed to solve these problems. Liu [11], Tao [12], Johannessen [13], Hanson [14], Zhang [15], Dørum [16], Ingold [17] and Ingold [18] proposed the use of coding region Single Nucleotide Polymorphism (SNP) on mRNA, which was helpful for assigning alleles to corresponding body fluids in mixtures analysis, but RNA degradation will limit its application [11–19]. Watanabe [20], Xie [21], Li [1] and Li [22] also found that the methylation-based SNP/Indel/ (Short Tandem Repeat) STR genotyping method was useful for confirming the presence of semen and genotyping the semen-specific DNA in body fluid mixtures [1,20–22]. However, these methods are based on methylation-based PCR or methylation-sensitive restriction enzyme-based PCR, which may generate amplicons from non-semen DNA. Moreover, some SNP/Indel-based loci have limited polymorphisms, and some STR-based loci have large amplicon sizes. Therefore, it is necessary to develop new composite genetic markers with higher polymorphisms and smaller amplicon sizes and to use more powerful technologies to obtain more information for the analysis of semen–vaginal fluid mixtures.

As proposed by Kidd [23], microhaplotype consists of at least two closely linked SNPs with at least three allele combinations [23]. Microhaplotype combines the advantages of SNP and STR, including more polymorphism than SNP and smaller amplicon size than STR [24]. These microhaplotype loci have been widely used in mixture analysis, but do not provide body fluid identification information [25–29]. However, our previous study indicated that there were microhaplotype sites in the neighbouring region of blood-specific CpG sites, which can form blood-specific methylation-microhaplotype loci that were useful for analysing blood-containing mixtures [30]. Meanwhile, the massively parallel sequencing (MPS) technology can simultaneously provide DNA methylation and genotype data, and perform multiplex amplification. Therefore, in this study, it is proposed to combine semen-specific methylation sites and nearby closely related microhaplotype sites to form a new composite genetic marker (semen-specific methylation-microhaplotype, S-MMH) and to detect it using MPS technology to confirm the presence of semen and link semen to a true donor in semen–vaginal fluid mixtures.

## 2. Material and methods

### 2.1. Selecting semen-specific methylation-microhaplotype loci

#### 2.1.1. Selecting semen-specific CpG sites

The keywords ‘DNA methylation’ and ‘semen’ were searched in the PubMed dataset, and studies using DNA methylation to identify body fluid types were summarized [1,7,20–22,31–61]. The semen-related CpG sites mentioned in studies were recorded. The median methylation levels of these CpG sites in common body fluids (including semen, blood, saliva and vaginal fluid) were then obtained after searching the consensus reference methylomes (CRMs) of the methylation bank (MethBank) (https://ngdc.cncb.ac.cn/methbank/v3/). Finally, the selection criteria for semen-specific CpG sites were set as follows: (i) the selected semen-specific CpG site was mentioned in at least one study using DNA methylation to identify body fluids; (ii) after searching the CRMs of the MethBank, the differences in median methylation levels of the selected semen-specific CpG sites between semen and non-semen (peripheral blood, saliva and vaginal fluid) had to be >80%.

#### 2.1.2. Selecting microhaplotype sites

After searching the genomic region adjacent to the selected semen-specific CpG sites (300 bp upstream or downstream), the appropriate microhaplotype sites were selected. The main criteria for the appropriate microhaplotype sites selected were as follows: (i) at least two SNPs with different allele frequencies were included in each microhaplotype site; (ii) each SNP within microhaplotype sites had the minor allelic frequency (MAF) >=0.05; (iii) based on Han Chinese in Beijing (CHB), each microhaplotype site had the effective number of alleles (Ae) >2.0; and (iv) each SNP within microhaplotype sites did not include the allele genotype C. Because the unmethylated C was converted to T after bisulfite conversion, it cannot be distinguished from the original allele genotype T.

#### 2.1.3. Combining into semen-specific methylation-microhaplotype loci

Ultimately, the candidate S-MMH loci consisted of a combination of selected semen-specific CpG sites and suitable closely related microhaplotype sites. The length of all S-MMH loci was smaller than 250 bp. According to the established criteria proposed by Kidd [62] for defining microhaplotype, this new composite genetic marker was named MMHxxZHAxxx [62]. Finally, six S-MMH loci were selected. But for one of the selected S-MMH loci, in order to make it easier to design the primers, we selected the CpG site located 76 bp upstream of the published semen-related CpG site as the semen-specific CpG site. This was because a previously published study by Watanabe [63] suggested that there may be more body fluid-specific CpG sites in neighbouring regions [63].

In addition, to further improve the power of discrimination, five semen-specific methylation-SNP (S-MS) loci consisting of one semen-specific CpG site and one SNP site were also included. The SNPs contained in five S-MS loci did not include the allele genotype C, the MAF of each SNP contained in S-MS loci was >=0.05, and the length of each S-MS locus was <250 bp. The nomenclature of S-MS loci was similar to that of S-MMH loci, for example MSxxZHAxxx.

### 2.2. Collecting samples and extracting DNA

A total of 63 samples from unrelated Chinese individuals, including 22 whole blood, 18 semen and 23 vaginal fluid samples, were collected. All study participants provided written informed consent. The HiPure Universal DNA Kit (Magen, China) was used to extract DNA of these samples, and the Qubit dsDNA HS Assay Kit (Thermo Fisher Scientific, USA) was used to quantify the concentrations of extracted DNA samples on the Qubit fluorometer.

### 2.3. Preparing semen–vaginal fluid mixtures

Two semen samples (J3523 and J3989) and two vaginal fluid samples (Y162 and Y82) were randomly selected to form two mixture groups. The extracted DNA of semen sample J3523 and the extracted DNA of vaginal fluid sample Y162 were mixed in five different mixing ratios (1:1, 1:3, 1:8, 5:1 and 10:1) to form the semen–vaginal fluid mixture group 1, named mixture 1, 3, 5, 8 and 10, respectively. The extracted DNA of semen sample J3989 and the extracted DNA of vaginal fluid sample Y82 were mixed in five different mixing ratios (1:1, 1:4, 1:9, 4:1 and 9:1) to form the semen–vaginal fluid mixture group 2, named mixture 2, 4, 6, 7 and 9, respectively.

### 2.4. Bisulfite conversion and multiplex amplification

In total, 100 ng DNA from 63 unrelated individuals and 10 mixture samples was converted using the EZ DNA Methylation-Gold™ Kit (Zymo Research, USA). The MethPrimer software (http://www.urogene.org/cgi-bin/methprimer2/MethPrimer.cgi) was used to design primers for each locus based on the converted DNA sequence. The primer sequences are listed in electronic supplementary material, table S1. The adaptor sequences added to the primers were as follows:

Forward adaptor: 5’- CCTACACGACGCTCTTCCGATCT-3’

Reverse adaptor: 5’- TTCAGACGTGTGCTCTTCCGATCT-3’

The multiplex amplification of selected loci was performed in a single reaction system (including converted DNA 8.5 µl, primer mix 4 µl and 2X Multiplex Mix 12.5 µl) using the KAPA 2G Rapid Multiplex PCR kit (KAPA Biosystems, USA). The multiplex amplification was executed under the following conditions: an initial denaturation temperature of 98℃ for 2 min; a denaturation temperature of 98℃ for 15 s, an annealing temperature of 60℃ for 4 min for a total of 27 cycles; and a final extension temperature of 72℃ for 10 min. The index PCR (including the above DNA products 10.5 µl, index primers 2 µl and 2X Multiplex Mix 12.5 µl) was then performed using the KAPA 2G Fast Multiplex PCR Kit (KAPA Biosystems, USA). The index PCR was executed under the following conditions: an initial denaturation temperature of 98℃ for 1 min; a denaturation temperature of 98℃ for 15 s, an annealing temperature of 60℃ for 30 s, an extension temperature of 72℃ for 30 s for a total of 18 cycles; and a final extension temperature of 72℃ for 1 min.

### 2.5. Massively parallel sequencing

**Figure 1.**
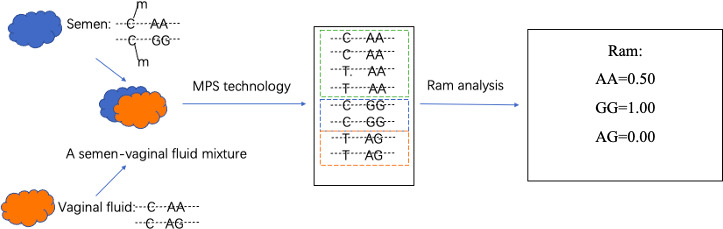
An example of R_am_ analysis in a semen–vaginal fluid mixture. (C-m presented the methylated status of target semen-specific CpG site within this locus, which remained C after Bisulfite conversion and sequencing; C presented the unmethylated status of target semen-specific CpG site within this locus, which converted to T after Bisulfite conversion and sequencing; AA, AG and GG indicated the genotypes of target microhaplotype site within this locus.)

The top strand of each locus was sequenced using the NovaSeq 6000 S4 Reagent Kit v1.5 (300 cycles) (Illumina, USA) on the Illumina NovaSeq system platform (Illumina, USA). Sequencing data were then filtered using Trimmomatic-0.38, and filtered reads were aligned to the human reference genome (hg19) using BSMAP (Version 2.74) [64,65]. Allele genotypes and methylation status (methylated or non-methylated) for each locus were available simultaneously for each sequencing read. After focusing on the allele genotypes of the target microhaplotype/SNP site within each locus, sequencing reads genotyped as the same allele could be clustered together, some of which were methylated and some of which were unmethylated at the closely linked target semen-specific CpG site. So, the allele-specific methylation rate (R_am_), which represents the ratio of methylated reads to the total number of clustered reads genotyped for a specific allele, could be calculated for each allele at each locus (shown in figure 1). After indirect assessment of bisulfite conversion by CHH methylation (H stands for any base except G), bisulfite conversion rate was confirmed to be greater than 99.2% for each sample. In addition, in order to perform reproducibility analysis, one randomly selected semen sample (J3523) and one randomly selected vaginal fluid sample (Y162) were extracted, transformed, amplified and sequenced a second time. Therefore, the allele genotypes and corresponding R_am_ values from 63 unrelated individual samples, 2 repeated samples, and 10 mixture samples were obtained and analysed.

### 2.6. Statistical analysis

#### 2.6.1. Body fluid specificity

Firstly, based on the CRMs of the MethBank, the median levels of methylation for each locus were obtained in different body fluids, including blood, saliva, vaginal fluid and semen. Then, to further verify the body fluid specificity of each locus, the mean values of R_am_ for each locus in different body fluids, including 22 whole blood, 18 semen and 23 vaginal fluid samples, were calculated using the SPSS version 20.0 software.

#### 2.6.2. Forensic parameters

The modified Powerstats version 1.2 software (https://www.promega.com.cn/products/genetic-identity/) was used to calculate allele frequencies, discrimination power (PD), observed heterozygosity (Ho), compound discrimination power (CPD) and Hardy–Weinberg equilibrium (HWE) for each locus based on the data of 63 unrelated Chinese individuals. Ae of each locus was also calculated based on the data of 63 unrelated Chinese individuals according to the formula proposed by Kidd [66].

#### 2.6.3. Analysis of semen–vaginal fluid mixtures

##### 2.6.3.1. Developing an allele categorization model

Three allele types are usually observed in semen–vaginal fluid mixtures, including the allele from semen-specific DNA (semen-specific DNA allele, S-allele), the allele from non-semen-specific DNA (non-semen-specific DNA allele, NS-allele) and the same allele shared between semen and non-semen (the same allele). For semen-specific hypermethylated loci of semen–vaginal fluid mixtures, the R_am_ of S-alleles presented greater values, the R_am_ of NS-alleles presented smaller values and the R_am_ of the same alleles presented intermediate values. Similarly, for semen-specific hypomethylated loci of semen–vaginal fluid mixtures, the R_am_ of S-alleles presented smaller values, the R_am_ of NS-alleles presented greater values and the R_am_ of the same alleles presented intermediate values. Thus, the allele types in semen–vaginal fluid mixtures could be inferred based on the R_am_ distributions, helping to analyse semen–vaginal fluid mixtures.

K-nearest neighbour algorithm (KNN) is a non-parametric and supervised learning classifier that calculates the distance between a new data point and each data point in the training dataset and then predicts the class of the new data point based on the class of the K nearest data points. With the advantages of being easy to implement, flexible and requiring no explicit training, the KNN has been used in forensic science for age classification, gender prediction, and so on [67,68]. Therefore, KNN may be suitable for developing an allele categorization model on the basis of the distributions of R_am_ values.

The training dataset used to develop the allele categorization model included the R_am_ values of S-alleles, the R_am_ values of NS-alleles and the R_am_ values of the same alleles. We obtained the R_am_ values of S-alleles and NS-alleles from 63 unrelated Chinese individuals. But the R_am_ values of the same alleles were obtained from a series of simulated mixtures constructed based on the theoretical R_am_ distributions of the same alleles in semen-containing mixtures with mixing ratios of 10:1, 9:1, 5:1, 4:1, 1:1, 1:3, 1:4, 1:8 and 1:9 (semen:non-semen). The simulation process was as follows: (i) firstly, semen and non-semen (blood, vaginal fluid) samples were randomly selected to create simulated semen-containing mixtures, which had the same alleles for each locus; (ii) then, according to the formula presented in electronic supplementary material, table S2, the R_am_ values for the simulated same alleles were calculated. Therefore, the ‘semen-specific DNA alleles’ section consisting of R_am_ values from all semen samples, the ‘non-semen-specific DNA alleles’ section consisting of R_am_ values from all non-semen samples (blood and vaginal fluid), and the ‘the same alleles’ section consisting of R_am_ values from simulated semen-containing mixtures were included in the training dataset. Details of the training dataset are presented in electronic supplementary material, table S3. The developed allele categorization model was evaluated using precision, recall, f1-score and accuracy values based on the training dataset and then was applied to predict the allele types of semen–vaginal fluid mixtures in order to confirm the presence of semen and link semen to a true donor.

##### 2.6.3.2. Confirming the presence of semen

After applying the developed allele categorization model, the allele types of semen–vaginal fluid mixtures were predicted. If some alleles in a semen–vaginal fluid mixture were identified as S-alleles and/or the same alleles, the presence of semen could be confirmed. However, if all alleles in a semen–vaginal fluid mixture were identified as NS-alleles, the presence of semen could not be confirmed. Finally, we analysed the accuracy of confirming the presence of semen in semen–vaginal fluid mixtures with different mixing ratios.

##### 2.6.3.3. Linking semen to a true donor

Firstly, the commonly used STR typing technology based on the capillary electrophoresis platform or microhaplotype typing technology based on the MPS platform could be used to find potential donors of semen–vaginal fluid mixtures. Then, on this basis, the new composite genetic marker proposed in our study could be used as a supplementary tool to link semen to a true donor.

Three types of alleles (S-allele, the same allele and NS-allele) were obtained in semen–vaginal fluid mixtures after application of the developed allele categorization model. Based on the prediction results, we could obtain a conservative model-predicted genotype including S-alleles and the same alleles, which was thought to be highly representative of the genotype of the semen donor. We then compared the genotypes of potential donors with the conservative model-predicted genotype and used the random man not excluded (RMNE) method to link semen to a true donor in semen–vaginal fluid mixtures.

The RMNE method calculates the likelihood that a random person is misidentified as being present in the mixture. The random person with the smaller likelihood is considered to be more likely to be a donor for the mixture. Referring to the improved RMNE method developed by Zhu [69], the calculation process in this study was as follows: (i) when the genotypes of potential donors at a locus matched the model-predicted genotype (i.e. alleles of the potential donor genotype at a locus were all included in alleles of the model-predicted genotype), we could calculate the RMNE value for this matched locus as follows:


$${P(RMNE)}_{MMH}={\left(\sum_{i=1}^{n}P_{i}\right)}^{2}$$

where n represented the alleles number of the model-predicted genotype at a locus, and $P_{i}$ represented the allele i frequency at a locus.

When the genotypes of potential donors at a locus did not match the model-predicted genotype (i.e. alleles of the potential donor genotype at a locus were not all included in alleles of the model-predicted genotype), we did not calculate the RMNE value for this mismatched locus; (ii) finally, we calculated the P(RMNE) value of a potential donor by cumulatively multiplying the P(RMNE)_MMH_ values of all matched loci. For each potential donor in a semen–vaginal fluid mixture, the corresponding P(RMNE) value was calculated, and the potential donor with a smaller P(RMNE) value was identified as the true semen donor for this semen–vaginal fluid mixture. Finally, we analysed the accuracy of linking semen to a true donor in semen–vaginal fluid mixtures with different mixing ratios.

## 3. Results

### 3.1. General information

Six S-MMH loci and five S-MS loci were selected. Details of these selected loci are shown in table 1. One locus consisted of three SNP sites and one CpG site, five loci consisted of two SNP sites and one CpG site, and five loci consisted of one SNP site and one CpG site. These 11 loci were separately located on 10 different chromosomes. The average size of these loci was 136.8 bp, and all amplicon sizes were smaller than 300 bp.

**Table 1.** Details of the selected loci.

locus name	Chr#	#SNPs	#CpGs	the included SNPs ID and CpGs ID	the position (hg19)	extent in bp
MS01ZHA088	1	1	1	cg25108325 rs2806529	64669473 64669567	95
MS02ZHA097	2	1	1	rs10490445 cg23521140	37617484 37617601	118
MMH04ZHA019	4	2	1	rs6816279 cg27598661 rs61910702	184404508 184404666 184404688	181
MS05ZHA087	5	1	1	rs3749787 cg17433294	151784183 151784252	70
MMH07ZHA093	7	2	1	cg05261336 rs56109702 rs4388364	1032905 1032919 1033045	141
MMH12ZHA005	12	2	1	rs79478437 rs4767507 cg05770241	117593387 117593391 117593501	115
MMH12ZHA094	12	2	1	rs3803100 cg11768416 rs3803099	91348640 91348697 91348786	147
MMH16ZHA089	16	3	1	rs825586 rs12596982 cg09245584+76 bp rs825584	87669882 87669951 87670005 87670042	161
MMH17ZHA059	17	2	1	cg22314465 rs1809240 rs4993559	72109759 72109874 72109948	190
MS19ZHA085	19	1	1	rs6509437 cg21292909	50059718 50059938	221
MS20ZHA086	20	1	1	rs6124050 cg27387705	59598779 59598844	66

Finally, among 63 unrelated individual samples, 2 repeated samples and 10 mixture samples, 9 loci were amplified and sequenced successfully, except for loci MMH04ZHA019 and MMH17ZHA059. As an example, the raw sequencing data of a semen-specific hypomethylated locus (MS05ZHA087) in a semen–vaginal fluid mixture with a mixing ratio of 1:1 (semen:vaginal fluid) from the semen–vaginal fluid group 2 is presented in electronic supplementary material, figure S1. The first black box represented the target SNP site within this locus and the second black box represented the target semen-specific CpG site within this locus. Reads genotyped as allele A of the target SNP site were clustered in the upper half of the figure, and C of the closely linked target semen-specific CpG site in these reads was converted to T. Reads genotyped as allele G of the target SNP site were clustered in the lower half of the figure, and C of the closely linked target semen-specific CpG site in these reads remained at C. So, the allele A was an unmethylated allele that could be inferred as an S-allele, while the allele C was a methylated allele that could be inferred as a NS-allele.

Moreover, the allele genotypes and corresponding R_am_ values from two repeated samples are listed in electronic supplementary material, table S4. The genotypes of nine successfully sequenced loci in the repeated semen sample were consistent with those of semen sample J3523, and the genotypes of nine successfully sequenced loci in the repeated vaginal fluid sample were also consistent with those of the vaginal fluid sample Y162. After comparing the duplicate samples with their original samples, the mean absolute difference in R_am_ was 0.02. These results suggested that these loci were well reproducible.

### 3.2. Body fluid specificity

Based on the CRMs of the MethBank, the median methylation levels of nine successfully sequenced loci in different body fluids (including blood, saliva, vaginal fluid and semen) are shown in table 2. For loci MS01ZHA088, MS05ZHA087, MMH12ZHA005, MMH12ZHA094, MS19ZHA085 and MS20ZHA086, the median levels of methylation in semen were lower than those in blood, saliva and vaginal fluid, with a difference of >80%. For loci MS02ZHA097, MMH07ZHA093 and MMH16ZHA089, the median levels of methylation in semen were higher than those in blood, saliva and vaginal fluid, with a difference of >80%.

**Table 2.** The median methylation levels of nine successfully sequenced loci in common body fluids based on the CRMs of the MethBank.

locus(CpGs ID)	blood	saliva	vaginal fluid	semen
MS01ZHA088(cg25108325)	0.93	0.93	0.92	0.04
MS02ZHA097(cg23521140)	0.04	0.02	0.02	0.96
MS05ZHA087(cg17433294)	0.97	0.98	0.98	0.02
MMH07ZHA093(cg05261336)	0.03	0.02	0.01	0.96
MMH12ZHA005(cg05770241)	0.98	0.99	0.99	0.02
MMH12ZHA094(cg11768416)	0.96	0.96	0.97	0.02
MMH16ZHA089(cg09245584)	0.06	0.05	0.03	0.96
MS19ZHA085(cg21292909)	0.94	0.98	0.99	0.01
MS20ZHA086(cg27387705)	0.91	0.98	0.97	0.01

**Figure 2.**
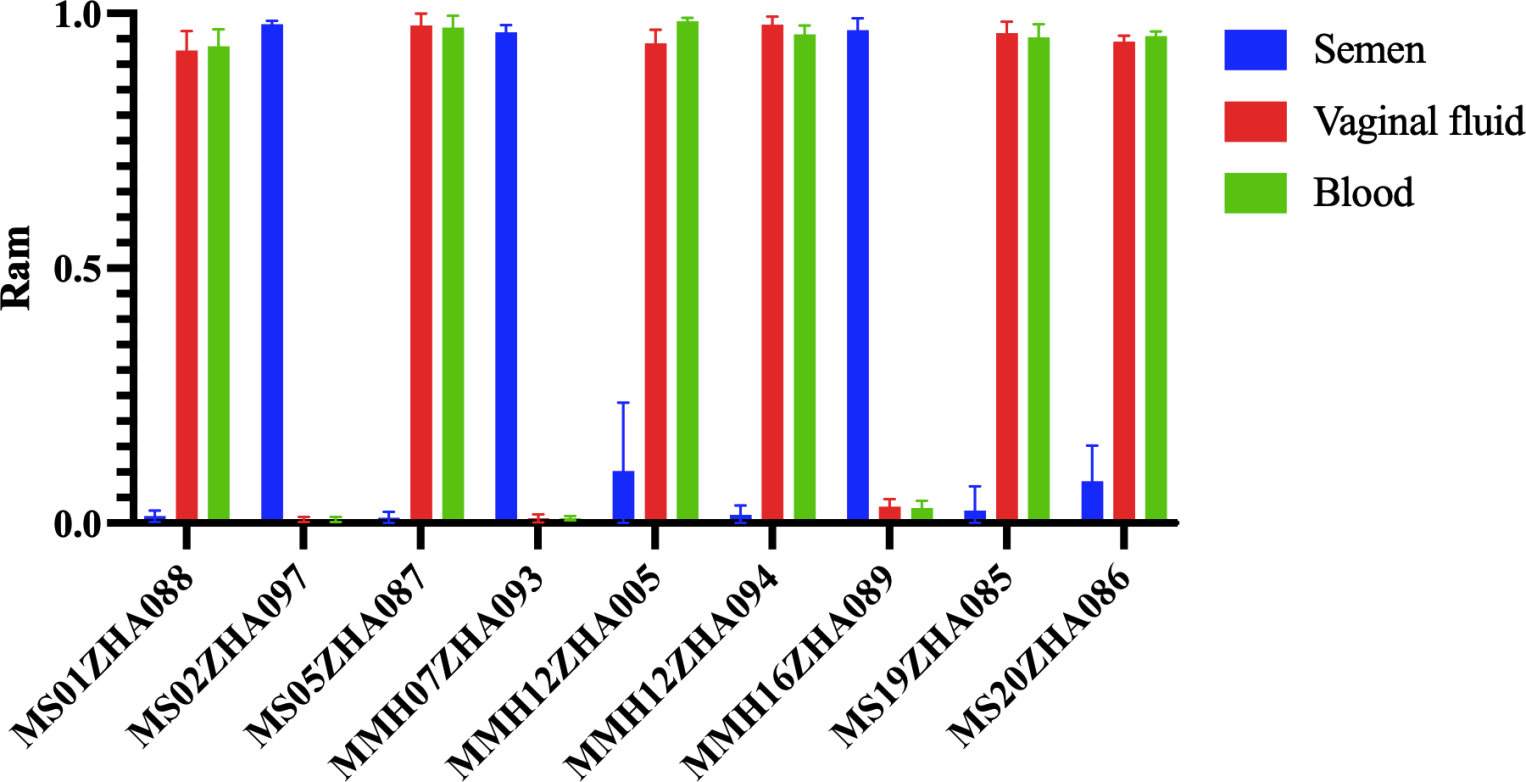
The comparison of mean R_am_ values at nine loci in 22 blood, 18 semen and 23 vaginal fluid samples.

Meanwhile, based on the data of 63 unrelated Chinese individuals, the mean R_am_ values of these nine loci in different body fluids (including blood, semen and vaginal fluid) are presented in figure 2. Similar to the above results, the loci MS01ZHA088, MS05ZHA087, MMH12ZHA005, MMH12ZHA094, MS19ZHA085 and MS20ZHA086 were semen-specific hypomethylated loci, while the loci MS02ZHA097, MMH07ZHA093 and MMH16ZHA089 were semen-specific hypermethylated loci.

Moreover, for the semen-specific CpG site within locus MMH16ZHA089, the median methylation levels of cg09245584 were queried in the CRMs of the MethBank, but the mean R_am_ values of the CpG site located 76 bp upstream of cg09245584 were analysed in actual samples. Cg09245584 and the CpG site located 76 bp upstream of cg09245584 were identified as the semen-specific CpG site. The results further confirmed Watanabe stated view that there may be more body fluid-specific CpG sites in neighbouring regions, which may be useful for the design of primers and the construction of methylation-microhaplotype loci.

### 3.3. Forensic parameters

The genotypes and R_am_ values of nine successfully sequenced loci of 63 unrelated Chinese individuals are presented in electronic supplementary material, table S5, and the forensic parameters of these loci are shown in table 3. The significant deviation from the HWE was not observed at nine loci after the Bonferroni correction (*p* < 0.05/9 = 0.006). A total of 28 alleles were obtained, and the CPD was 0.9999750. The Ho values were in the range of 0.33 (MS19ZHA085)–0.73 (MMH16ZHA089), and the mean Ho value was 0.50. The Ae values were in the range of 1.59 (MS19ZHA085)–4.02 (MMH16ZHA089), with a mean Ae value of 2.24. Meanwhile, as presented in electronic supplementary material, figure S2, the Ae values of methylation-microhaplotype loci were higher than those of methylation-SNP loci, indicating the methylation-microhaplotype loci had stronger discrimination power.

**Table 3.** The forensic parameters of nine successfully sequenced loci based on the data of 63 unrelated Chinese individuals.

MS01ZHA088		MS02ZHA097		MS05ZHA087		MMH07ZHA093			
A	0.63	T	0.43	A	0.37	AG	0.21		
T	0.37	G	0.57	G	0.63	GT	0.57		
						GG	0.22		
PD	0.63	PD	0.66	PD	0.61	PD	0.75		
Ho	0.33	Ho	0.35	Ho	0.46	Ho	0.63		
HWE	0.03	HWE	0.02	HWE	0.86	HWE	0.43		
Ae	1.88	Ae	1.96	Ae	1.88	Ae	2.39		

MMH12ZHA005		MMH12ZHA094		MMH16ZHA089		MS19ZHA085		MS20ZHA086	
AG	0.43	AT	0.2	AAA	0.35	A	0.25	A	0.33
GA	0.56	GT	0.51	AAG	0.02	G	0.75	G	0.67
GG	0.02	GG	0.29	ATG	0.02				
				AGA	0.01				
				AGG	0.25				
				GAA	0.02				
				GTG	0.01				
				GGA	0.22				
				GGG	0.11				
PD	0.64	PD	0.74	PD	0.89	PD	0.54	PD	0.57
Ho	0.52	Ho	0.7	Ho	0.73	Ho	0.33	Ho	0.49
HWE	0.84	HWE	0.21	HWE	0.62	HWE	0.51	HWE	0.43
Ae	2.03	Ae	2.61	Ae	4.02	Ae	1.59	Ae	1.78

### 3.4. Analysis of semen–vaginal fluid mixtures

#### 3.4.1. Developing an allele categorization model

According to the training dataset, an allele categorization model was developed using KNN. As presented in electronic supplementary material, table S6, the ranges of precision, recall and f1-score values of the allele categorization model based on the training dataset were 0.75−1.00, 0.50−1.00 and 0.60−1.00, respectively, and the mean precision, recall and f1-score values were 0.94, 0.92 and 0.92, respectively. The accuracy values for nine loci based on the training dataset were all larger than 0.80, with an average accuracy value of 0.94.

Then, the allele types in semen–vaginal fluid mixtures were predicted by the developed allele categorization model. The accuracy of allele type identification in semen–vaginal fluid mixtures with different mixing ratios is listed in table 4. A total of 164 alleles were observed, of which 27 allele types were incorrectly identified. Six S-alleles were misidentified as the same alleles. Six NS-alleles were misidentified as the same alleles. Two same alleles were misidentified as S-alleles, and 13 same alleles were misidentified as NS-alleles. The total accuracy value for nine loci in semen–vaginal fluid mixtures was 0.84. These results suggested that the allele categorization model developed using KNN had a good ability to predict the allele types in semen–vaginal fluid mixtures.

**Table 4.** The accuracy of allele type identification in semen–vaginal fluid mixtures with different mixing ratios.

mixture names	mixture groups	mixing ratios	total numbers of alleles incorrectly identified	semen-specific DNA alleles incorrectly identified as the same alleles (numbers)	the same alleles incorrectly identified as semen-specific DNA alleles (numbers)	non-semen-specific DNA alleles incorrectly identified as the same alleles (numbers)	the same alleles incorrectly identified as non-semen-specific DNA alleles (numbers)
mixture 1	semen–vaginal fluid mixture group 1	semen:vaginal = 1:1	3	1	0	2	0
mixture 2	semen–vaginal fluid mixture group 2	semen:vaginal = 1:1	0	0	0	0	0
mixture 3	semen–vaginal fluid mixture group 1	semen:vaginal = 1:3	3	1	0	0	2
mixture 4	semen–vaginal fluid mixture group 2	semen:vaginal = 1:4	6	3	0	0	3
mixture 5	semen–vaginal fluid mixture group 1	semen:vaginal = 1:8	5	1	0	0	4
mixture 6	semen–vaginal fluid mixture group 2	semen:vaginal = 1:9	4	0	0	0	4
mixture 7	semen–vaginal fluid mixture group 2	semen:vaginal = 4:1	3	0	0	3	0
mixture 8	semen–vaginal fluid mixture group 1	semen:vaginal = 5:1	1	0	1	0	0
mixture 9	semen–vaginal fluid mixture group 2	semen:vaginal = 9:1	1	0	0	1	0
mixture 10	semen–vaginal fluid mixture group 1	semen:vaginal = 10:1	1	0	1	0	0
totals			27	6	2	6	13

#### 3.4.2. Confirming the presence of semen

The allele types in semen–vaginal fluid mixtures could be identified after applying the developed allele categorization model, and the presence of semen could be confirmed if some alleles in a semen–vaginal fluid mixture were identified as S-alleles and/or the same alleles. The R_am_ distributions and prediction results of nine loci in semen–vaginal fluid mixtures with different mixing ratios are shown in electronic supplementary material, table S7. The accuracy of confirming the presence of semen in semen–vaginal fluid mixtures with different mixing ratios is presented in table 5. The presence of semen was confirmed in all 10 mixture samples, which indicated that these loci had a good ability to confirm the presence of semen in semen–vaginal fluid mixtures with mixing ratios of 1:1, 1:3, 1:4, 1:8, 1:9, 4:1, 5:1, 9:1 and 10:1 (semen:vaginal fluid).

**Table 5.** The accuracy of confirming the presence of semen in semen–vaginal fluid mixtures with different mixing ratios.

mixture names	mixture groups	mixing ratios	identification of the presence of semen
mixture 1	semen–vaginal fluid mixture group 1	semen:vaginal = 1:1	YES
mixture 2	semen–vaginal fluid mixture group 2	semen:vaginal = 1:1	YES
mixture 3	semen–vaginal fluid mixture group 1	semen:vaginal = 1:3	YES
mixture 4	semen–vaginal fluid mixture group 2	semen:vaginal = 1:4	YES
mixture 5	semen–vaginal fluid mixture group 1	semen:vaginal = 1:8	YES
mixture 6	semen–vaginal fluid mixture group 2	semen:vaginal = 1:9	YES
mixture 7	semen–vaginal fluid mixture group 2	semen:vaginal = 4:1	YES
mixture 8	semen–vaginal fluid mixture group 1	semen:vaginal = 5:1	YES
mixture 9	semen–vaginal fluid mixture group 2	semen:vaginal = 9:1	YES
mixture 10	semen–vaginal fluid mixture group 1	semen:vaginal = 10:1	YES

However, the presence of semen could not be confirmed at two loci in mixtures 3 and 4, respectively. Similarly, the presence of semen could not be confirmed at three loci in mixture 5, and at six loci in mixture 6. The number of loci where the presence of semen could not be confirmed gradually increased as the percentage of semen components in semen–vaginal fluid mixtures decreased. The main reasons for this situation were the dropout of S-alleles or the misidentification of the same alleles as NS-alleles.

#### 3.4.3. Linking semen to a true donor

**Figure 3.**
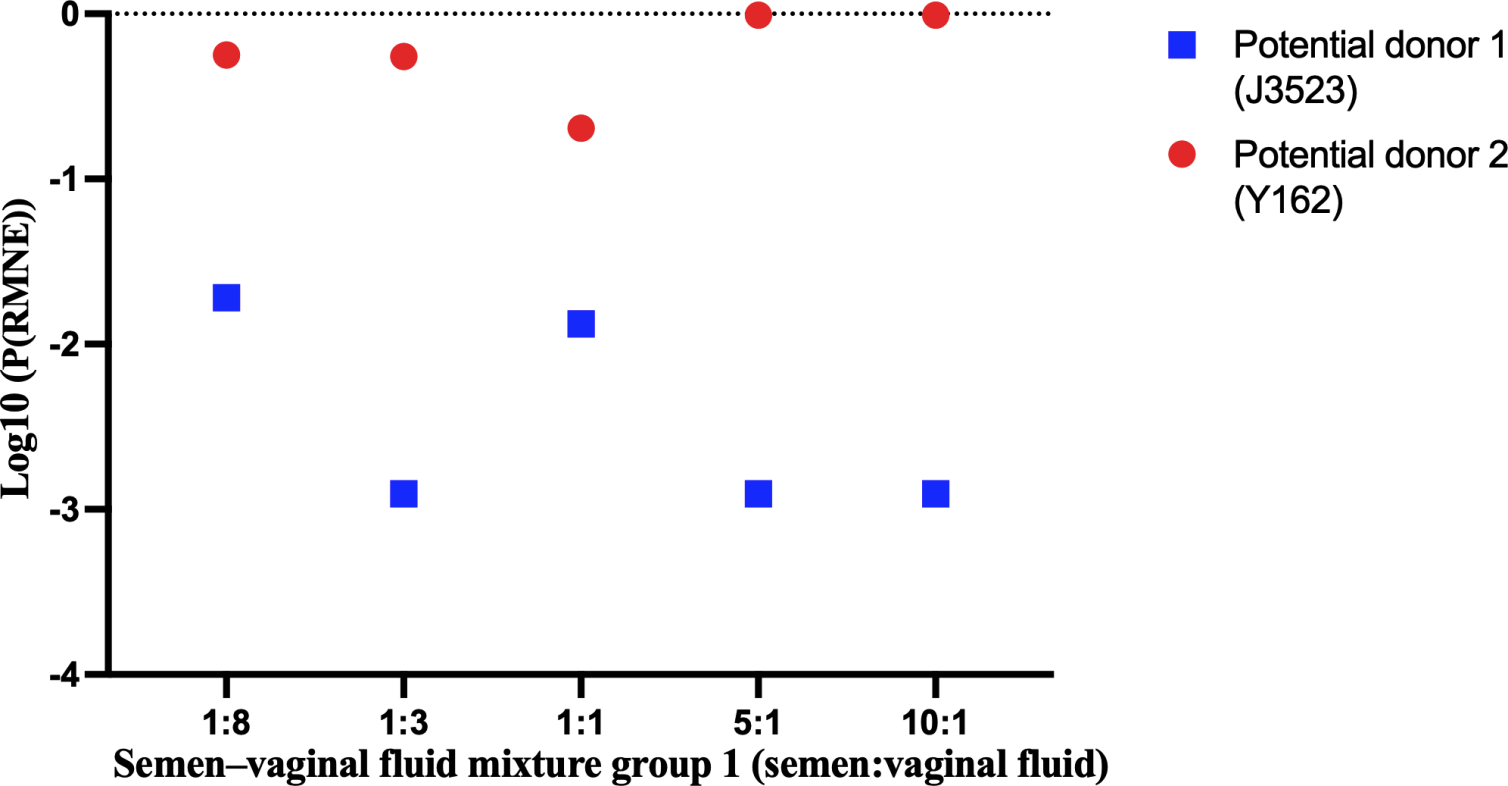
The Log10(P(RMNE)) values (Log10 values of P(RMNE)) of potential donors in semen–vaginal fluid mixtures from group 1.

**Figure 4.**
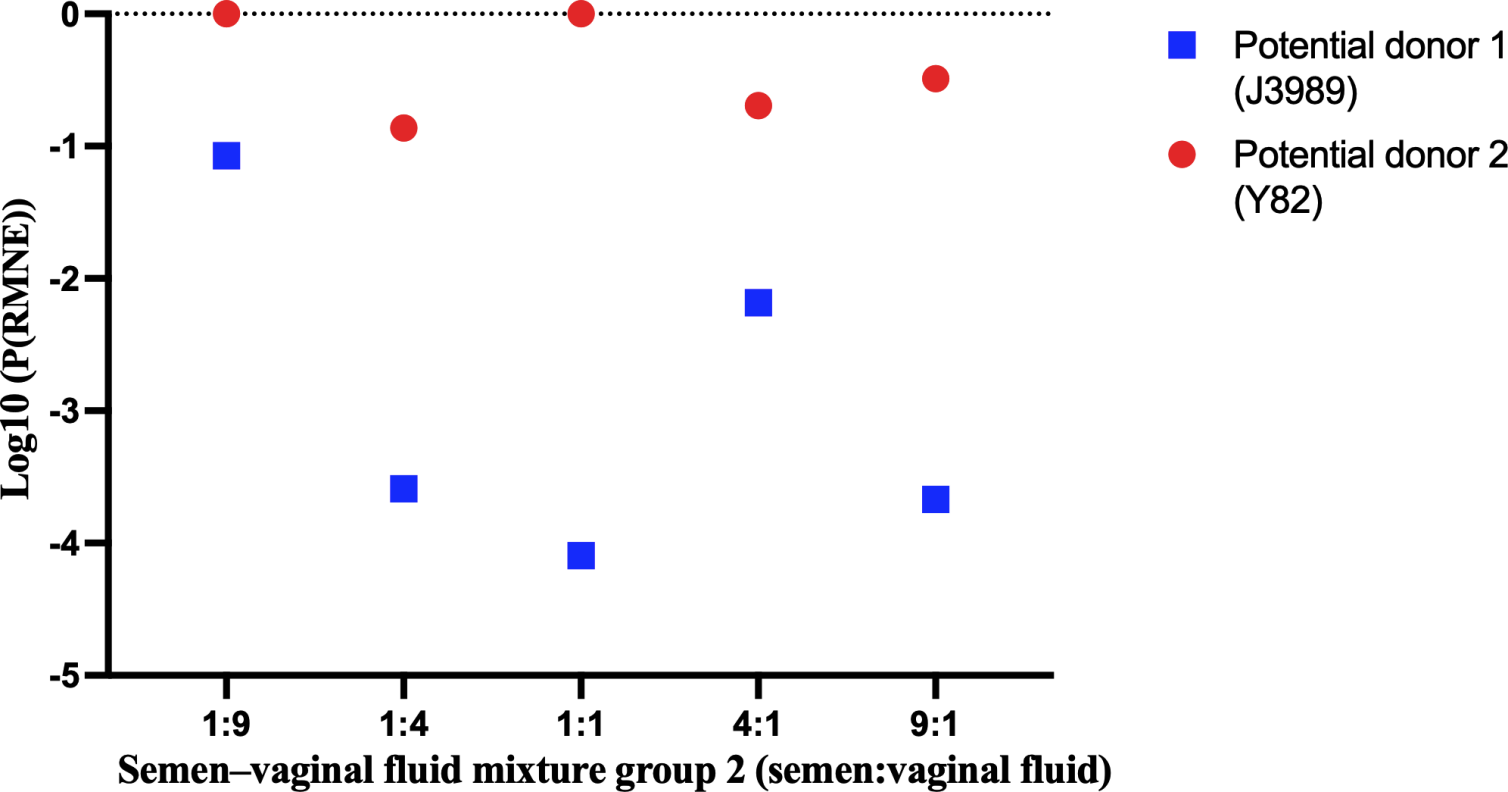
The Log10(P(RMNE)) values (Log10 values of P(RMNE)) of potential donors in semen–vaginal fluid mixtures from group 2.

Typically, the commonly used STR typing technology based on the capillary electrophoresis platform or microhaplotype typing technology based on MPS platform could find the potential donors of semen–vaginal fluid mixtures. On this basis, the semen could be linked to the true donor in semen–vaginal fluid mixtures after comparing the genotypes of potential donors with the conservative model-predicted genotype and using the RMNE method. The P(RMNE) values of potential donors in semen–vaginal fluid mixtures with different mixing ratios are listed in electronic supplementary material, table S8. For semen–vaginal fluid mixtures with different mixing ratios, the P(RMNE) values for semen donors ranged from 8.05E−05 to 8.45E−02, whereas the P(RMNE) values for vaginal fluid donors ranged from 1.37E−01 to 1.00E+00. The P(RMNE) values of semen donors were less than 0.10, whereas those of vaginal fluid donors were more than 0.10. Then, the Log10 values of P(RMNE) (Log10(P(RMNE))) for potiental donors were calculated and compared. A potential donor with a smaller Log10(P(RMNE)) value was considered to be the true donor to semen. As shown in figures 3 and 4, the Log10(P(RMNE)) values of semen donors (J3523 and J3989) were smaller than those of vaginal fluid donors (Y162 and Y82), which indicated that utilization of these loci in semen–vaginal fluid mixtures with mixing ratios of 1:1, 1:3, 1:4, 1:8, 1:9, 4:1, 5:1, 9:1 and 10:1 (semen:vaginal fluid) could correctly link semen to the true donor.

## 4. Discussion

In this study, in order to confirm the presence of semen and link semen to a true donor in semen–vaginal fluid mixtures, semen-specific CpG sites and closely related microhaplotype sites formed a new composite genetic marker (S-MMH) and detected it using the MPS technology. According to the selected criteria, six S-MMH loci were selected. In addition, to further improve the power of discrimination, five S-MS loci were also included. Finally, except for loci MMH04ZHA019 and MMH17ZHA059, the other nine loci were successfully amplified and sequenced. The failure of amplification and sequencing of these two loci is due to the inability to design suitable multiplex amplification primers.

For the nine successfully sequenced loci, there were three semen-specific hypermethylated loci and six semen-specific hypomethylated loci, which had the ability to distinguish semen from non-semen. These nine loci were only investigated in actual blood, semen and vaginal fluid samples in our study due to the time and funding constraints of sample collection. In the future, these loci should also be investigated in other frequently obtained forensic samples (such as saliva and skin) to further clarify the body fluid specificity.

Based on the data of 63 unrelated Chinese individuals, the CPD of nine successfully sequenced loci was 0.9999750. But the CPD of these loci was less than 0.999999999. The Ae values of methylation-microhaplotype loci were higher than those of methylation-SNP loci, indicating the methylation-microhaplotype loci had a stronger power of discrimination. There are several main reasons for the smaller numbers and lower information contents of semen-specific methylation-microhaplotype/SNP loci included in our study. Firstly, only a limited number of semen-specific CpG sites have suitable microhaplotype/SNP sites in nearby regions; secondly, a small number of SNP sites are included because all methylation-microhaplotype loci included in our study are less than 300 bp in length; and lastly, SNPs containing the C alleles are excluded. However, these nine loci had a certain power of discrimination, which helped to narrow down the scope of forensic investigations. So, these loci could be used as supplementary tools to commonly used genetic markers (STR, microhaplotype, etc.) for confirming the presence of semen and linking semen to a true donor in semen–vaginal fluid mixtures.

These loci in our study had a good ability to confirm the presence of semen and link semen to a true donor in semen–vaginal fluid mixtures with mixing ratios of 1:1, 1:3, 1:4, 1:8, 1:9, 4:1, 5:1, 9:1 and 10:1 (semen:vaginal fluid). However, in the course of parsing the results, we found the occurrence of both allele dropout and allele type misidentification. Theoretically, 10 mixture samples had a total of 190 alleles. After analysing the sequencing data, 164 alleles were observed, but 26 alleles dropped out, including 14 S-alleles and 12 NS-alleles. The dropout numbers of S-alleles gradually increased with decreasing percentage of semen components in semen–vaginal fluid mixtures. Similarly, the dropout numbers of NS-alleles gradually increased with decreasing percentage of non-semen components in semen–vaginal fluid mixtures. In total, 27 allele types were also incorrectly identified. The dropout of S-alleles can affect the ability of loci to confirm the presence of semen and the values of potential semen donors. Misidentifying NS-alleles as the same alleles can lead to smaller differences in values between potential semen donors and vaginal fluid donors. Thus, it may be difficult to confirm the presence of semen and to link semen to a true donor when there is are large numbers of allele dropout and allele type misidentification in semen–vaginal fluid mixtures.

Allele dropout is mainly caused by the further destruction of minor components of mixtures after bisulfite conversion. In the future, it is expected that the numbers of allele dropout will be reduced by using gentler conversion methods and improving multiplex amplification systems, making it possible to analyse extremely unbalanced semen–vaginal fluid mixtures with a very small proportion of semen components using this new composite genetic marker (S-MMH). Currently, a small number of studies have attempted to detect DNA methylation using bisulfite-free conversion methods, which may help address the problem of allele dropout [70,71].

In several previous studies, KNN can be used to accurately solve categorization problems such as age and gender grouping [67,68]. Based on these findings, in this study, we made a preliminary attempt to develop an allele categorization model using KNN and applied it to predict allele types in semen–vaginal fluid mixtures. Except for locus MMH12ZHA005, the accuracy based on the training dataset was greater than 0.90 for the remaining eight loci. The lower accuracy for locus MMH12ZHA005 is mainly due to the presence of allele-specific methylation. For semen samples, the R_am_ values of allele AG at this locus were in the range of 0.02−0.50, while the R_am_ values of allele GA at this locus were in the range of 0.00−0.07. In the future, the accuracy of the allele categorization model in confirming allele types in semen–vaginal fluid mixtures could be further improved by using more advanced and refined Artificial Intelligence (AI) algorithms and by avoiding the selection of loci where allele-specific methylation is present.

Furthermore, in this study, we only investigated semen–vaginal fluid mixtures containing DNA from two individuals, which were most common in sexual assault cases. Although it is theoretically possible to predict all S-alleles (whether they contain one or two individuals) in semen–vaginal fluid mixtures using the allele categorization model developed on the basis of this new composite genetic marker (S-MMH), the ability of these loci to confirm the presence of semen and link semen to a true donor should also be explored in the future in semen–vaginal fluid mixtures that contain DNA from multiple individuals. Based on available DNA conversion and amplification techniques, the S-MMH loci have the ability to confirm the presence of semen and link semen to a true donor in semen–vaginal fluid mixtures with mixing ratios of 1:1, 1:3, 1:4, 1:8, 1:9, 4:1, 5:1, 9:1 and 10:1 (semen:vaginal fluid). However, the efficacy of these loci in more extreme unbalanced mixtures still warrants further exploration in the future.

## 5. Conclusion

Therefore, when encountering suspected semen–vaginal mixtures containing small amounts of semen or without intact cells in actual forensic cases, although differential extraction strategy and laser capture microscopy technology may not come in handy, the potential donors of these semen–vaginal mixtures can be first identified by STR or microhaplotype typing techniques, and then this new composite genetic marker (S-MMH) can be used as a supplementary tool for confirming the presence of semen and linking semen to a true donor. However, the present study is only a proof-of-concept study aimed at analysing the application of S-MMH loci in semen–vaginal fluid mixtures, and important influencing factors such as allele dropout and allele type misidentification still deserve an in-depth investigation in the future.

## Data Availability

All data generated or analysed during this study are included in this published article and its supplementary information files [72].
